# Effects of serotonin on skeletal muscle growth

**DOI:** 10.1186/1753-6561-6-S3-O3

**Published:** 2012-06-01

**Authors:** Suchismita Chandran, Tingqing Guo, Teresa Tolliver, Weiping Chen, Dennis L Murphy, Alexandra C McPherron

**Affiliations:** 1 Genetics of Development and Disease Branch, National Institute of Diabetes and Digestive and Kidney Diseases, NIH, Bethesda, MD 20892, USA; 2 Laboratory of Clinical Science, National Institute of Mental Health, NIH, Bethesda, MD 20892, USA; 3 Microarray Core Facility, National Institute of Diabetes and Digestive and Kidney Diseases, NIH, Bethesda, MD 20892, USA

## Background

Myostatin (MSTN), a member of the transforming growth factor beta family, negatively regulates skeletal muscle mass. Deletion of the *Mstn* gene results in increased muscle mass. Also, *Mstn*-/- mice and mice expressing a dominant negative MSTN receptor (activin receptor type IIB, ACVR2B) show decreased adipose tissue. *Mstn*-/- mice fed a high fat diet gain less weight, have improved glucose tolerance and are more sensitive to insulin when compared to wild type controls. We are interested in characterizing molecules that may be downstream of MSTN signaling. We identified serotonin as a potential molecule that may interact with MSTN in skeletal muscle. Serotonin is thought to regulate both metabolism and cardiac hypertrophy, but it is not clear if serotonin is synthesized and functions in the skeletal muscle. We hypothesize that serotonin regulates skeletal muscle mass and metabolism, and the serotonin and myostatin pathways interact with each other.

## Materials and methods

Microarray screening was carried out on gastrocnemius muscle from transgenic mice carrying dominant negative ACVR2B and wild type littermates. Selected results were validated by real time qPCR (RT-qPCR) using cDNA from transgenic muscle and muscle from mice injected with a MSTN inhibitor (a soluble ACVR2B). Rat L6 myoblasts were differentiated in horse serum with or without serotonin. Myotube size was determined from anti-myosin heavy chain immunofluorescence using NIC Elements software (Nikon). Serotonin concentrations in transgenic muscle and L6 cells were determined by HPLC.

## Results

Expression of tryptophan hydroxylase 1 (Tph1), the enzyme that catalyzes the rate limiting step in serotonin synthesis was significantly increased by microarray in transgenic mice and by RT-qPCR in mice receiving the soluble receptor. Serotonin significantly promoted longitudinal growth of skeletal muscle fibers *in vitro.* Increased branching, differentiation and fusion of myoblasts into myotubes were also observed. Serotonin was detectable in muscle from transgenic mice and in L6 myoblasts and myotubes.

## Conclusions

This study is the first to show that serotonin is found in skeletal muscle, and promotes muscle growth *in vitro.* We also show that the *Tph1* enzyme is expressed in skeletal muscle, and that *Tph1* expression increases with hypertrophy after MSTN inhibition. To elucidate the mechanism by which MSTN and serotonin pathways interact with each other, and their role in muscle growth and insulin signaling, we are using specific agonists and antagonists along with RT-qPCR to determine which of the 15 serotonin receptors are expressed by skeletal muscle fibers in presence or absence of MSTN. Since *Mstn*-/- mice show improved glucose tolerance, and serotonin has previously been implicated in insulin secretion and glucose uptake, we are interested in understanding how MSTN and serotonin may regulate glucose metabolism in skeletal muscle. We hope that this study will not only shed light on normal muscle development and metabolism, but will also suggest potential targets that could be manipulated therapeutically with respect to muscle wasting diseases, diabetes, and obesity.

